# 3D-printed TCP-HA scaffolds delivering MicroRNA-302a-3p improve bone regeneration in a mouse calvarial model

**DOI:** 10.1038/s41405-023-00177-1

**Published:** 2023-11-24

**Authors:** Pirawish Limlawan, Numpon Insin, Laurine Marger, Mélanie Freudenreich, Stéphane Durual, Anjalee Vacharaksa

**Affiliations:** 1 Department of Oral Medicine, Faculty of Dentistry, Chulalongkorn University, Pathumwan, Bangkok 10330 Thailand; 2 Research Unit on Oral Microbiology and Immunology, Faculty of Dentistry, Chulalongkorn University, Pathumwan, Bangkok 10330 Thailand; 3 Department of Chemistry, Faculty of Science, Chulalongkorn University, Pathumwan, Bangkok 10330 Thailand; 4 Biomaterials Laboratory, Division of Fixed Prosthodontics and Biomaterials, University Clinics of Dental Medicine, University of Geneva, 1 Rue Michel Servet, 1204 Geneva, Switzerland; 5 Department of Microbiology, Faculty of Dentistry, Chulalongkorn University, Pathumwan, Bangkok 10330 Thailand; 6 Master of Science Program in Geriatric Dentistry and Special Patients Care, Faculty of Dentistry, Chulalongkorn University, Pathumwan, Bangkok 10330 Thailand

**Keywords:** Dental biomaterials, Dental implants

## Abstract

**Objective:**

To demonstrate hydroxyapatite nanoparticles modified with cationic functional molecules. 3-aminopropyltriethoxysilane (HA-NPs-APTES) carrying microRNA-302a-3p (miR) in the 3D-printed tricalcium phosphate/Hydroxyapatite (TCP/HA) scaffold can increase healing of the critical-sized bone defect.

**Materials and methods:**

3D-printed TCP/HA were modified with HA-NPs-APTES by two methods (M1, M2). The dispersion of particles was visualized by fluorescent microscopy. Biocompatibility of the scaffolds was tested by alizarin assay. Delivery of miR to the cells and osteogenic gene expression were evaluated by qPCR. After selecting best method (M2), scaffolds, scaffolds+HA-NPs-APTES with or without miR were implanted in 4 mm mouse calvarium defect (*n* = 4 per group). After 2,4 and 6 weeks, bone regeneration were evaluated by microCT and histology sections.

**Results:**

Both M1 and M2 scaffolds were biocompatible with cell adhesion on its surface. M2 scaffold showed significant increase of miR, suggesting successful delivery, resulted in downregulation of its target mRNA COUP-TFII, and upregulation of RUNX2 mRNA. Calvarium defect with M2 scaffold also showed significantly higher BV/TV and higher number of filled spaces at all time points. Histomorphometry demonstrated new bone formed at the center of the HA-NPs-APTES-miR scaffold earlier than controls.

**Conclusion:**

TCP/HA scaffold modified with HA-NPs-APTES facilitated delivery of miR and enhanced bone regeneration.

## Introduction

Dentoalveolar bone defects resulting from trauma, infection, inflammation, or surgical procedures such as tumor removal may be too large to heal spontaneously. Restored anatomic bone shape and volume are necessary to support prostheses or dental implants and then regain functions and esthetics. Treatment options including bone grafts, or the combination of stem cells and 3-dimensional bone scaffold are proposed [1]. Among different bone grafting materials, autografts and allografts are commonly used due to high regenerative abilities combined to low immunogenicity. However, allografts are expensive, and the availability of sufficient bone tissue for autografting remains to be a significant challenge. Xenografts are also largely employed despite putative adverse antigenic responses and disease transmission [2].

Synthetic bone substitutes were developed to overcome these limitations and present good outcomes [3, 4]. Several studies showed that block bone substitute present better capacities to repair craniofacial defects when compared to particulates [5, 6] and suggest beneficial effects of using pre-shaped scaffolds [7]. In addition, by using 3D printing, defects volumes may be easily duplicated to produce personalized scaffolds based on patient volumetric imaging such as computed tomography (CT) and magnetic resonance imaging [8].

Most of the synthetic bone substitutes are made of calcium phosphate derivates that can be 3D-printed into personalized grafts [9] and implanted into host jawbone without adverse effects [10]. Biphasic calcium phosphates represent a combination that is structurally, biologically and mechanically highly efficient [11]. By mixing hydroxyapatite (HA) that confers high mechanical resistance [12] to tricalcium phosphate (TCP) which is highly resorptive and provides calcium- and phosphorus-rich microenvironment to promote bone regeneration, it is possible to guarantee anatomic volume maintaining and resorption for the benefit of host bone synthesis [13]. A mixture of 70-80% TCP and 20-30% HA may facilitate angiogenesis [14] and induce equivalent bone regeneration to that obtained with autografts [15].

Bone regeneration improvement can also be done through the addition of bioactive molecules that increase cell migration and attachment to finally promote osseous and vascular formation inside the scaffold [16]. Numerous growth factors including Bone morphogenetic protein 2 (BMP2), Vesicular endothelial growth factor (VEGF) or Fibroblast growth factor (FGF) are known potent activators of bone- and vascular formation [17]. Post-transcriptional regulators such as microRNAs (miRNAs) may be also excellent candidates [18]. In that respect, miRNA-302a-3p was reported to stimulate osteoblast functions by down-regulating COUP-TFII expression [19], a potent (i) repressor of osteogenic gene Runt-related transcription factor 2 (RUNX2) [20] and (ii) activator of receptor activator of nuclear factor kappa-beta ligand (RANKL) [21] which is essential for osteoclastogenesis [22]. MiRNAs are fragile and highly sensible to nuclease degradation [23]. Nanocarriers support, e.g. Hydroxyapatite nanoparticles (HA-NPs), help in stabilizing and delivering miRNA to target cells [24, 25]. HA-NPs are positively charged by calcium ions and attract the negative phosphate backbone from nucleotides [26]. In addition, HA-NPs provide several advantages as a delivery system including easy fabrication, adequate surface area for condensing nucleotides, biocompatibility, and osteoconductive properties [27]. After surface modification with 3-aminopropyltriethoxysilane (APTES), the surface charge was increased, this improves miRNA condensation and facilitates HA-NPs uptake [25]. Once combined with miRNA-302a-3P, HA-NPs-APTES succeeded in improving bone cell differentiation and mineralization in vitro [20]. The present work aimed at developing a TCP/HA scaffold that could deliver miRNA-302a-3P fixed on HA-NPs-APTES in vivo to stimulate bone regeneration in a mice calvaria model.

## Materials and methods

The experiment design illustration is provided in the supplementary file.

### In vitro experiments

#### Preparation of Hydroxyapatite nanoparticles modified with 3-aminopropyltriethoxysilane (HA-NPs-APTES)

Hydroxyapatite nanoparticles (HA-NPs) synthesis and surface modification by APTES were described previously [25]. Briefly, HA-NPs were prepared by mixing calcium nitrate solution (0.25 M) and phosphate solution (0.15 M) in the pH of 10 then, hydrothermally treated at 120 °C for 10 h in a Teflon-lined autoclave and then water-cooled to room temperature. The HA-NPs were collected by centrifugation, washed with deionized water, and subsequently dried overnight. To modify surface with APTES, 0.2 g HA-NPs were resuspended in 20 mL of 2.5% v/v APTES in anhydrous toluene at room temperature for 3 h. Then, nanoparticles were collected by centrifugation, and washed with toluene to remove excess APTES. The HA-NPs-APTES nanoparticles were dried at 60 °C for 24 h before further use. All the chemicals were purchased from Sigma-Aldrich, USA.

For fluorescein isothiocyanate (FITC) labeling, 50 mg HA-NPs-APTES were resuspended and stirred for 24 h in a solution of FITC-ethanol at 0.2 mg/mL (Merck KGaA, Darmstadt, Germany). Resulting FITC-tagged HA-NPs-APTES were then collected by centrifugation, washed with ethanol, and dried at 60 °C for 24 h.

#### 3D-printed TCP/HA scaffold surface modification with HA-NPs-APTES and miRNA

TCP/HA cement (Plotter-Paste-CPC, Innoterre gmbH, Radebeul, Germany) was extruded on a 3D discovery printer from regenHU (Villaz St Pierre, Switzerland) at a pression of 1 Bar, room temperature [7, 28–30]. Cylindrical scaffolds were made of 4 orthogonal layers of 250 µm rods with an inter-rod space of 200 µm. General procedure for TCP/HA scaffolds hardening was performed by immersion in sterile water for 48 h followed by air drying.

Two surface modification methods were applied during cement hardening.

First method (M1): The suspension for cement hardening was prepared by mixing 50 µg/ml HA-NPs-APTES with 5 nM miRNA-302a-3p in 10 µl of RNase free water for 10 min then 990 µl of sterile water was added. The printed TCP/HA cement was directly immersed for 48 h in this suspension to allow hardening.

Second method (M2): The general procedure was applied, and sterile water was removed after 48 h. Ten µl of HA-NPs-APTES 50 µg/ml and miRNA-302a-3p 5 nM were then dropped on scaffolds surface and left to dry for an additional 48 h.

Alternatively, some scaffolds were directly conjugated with miRNA (S-mi) without HA-NPs-APTES by hardening the printed scaffolds in sterile water with 5 nM miRNA-302a-3p for 48 h to allow for complete setting.

**Fig. 1:**
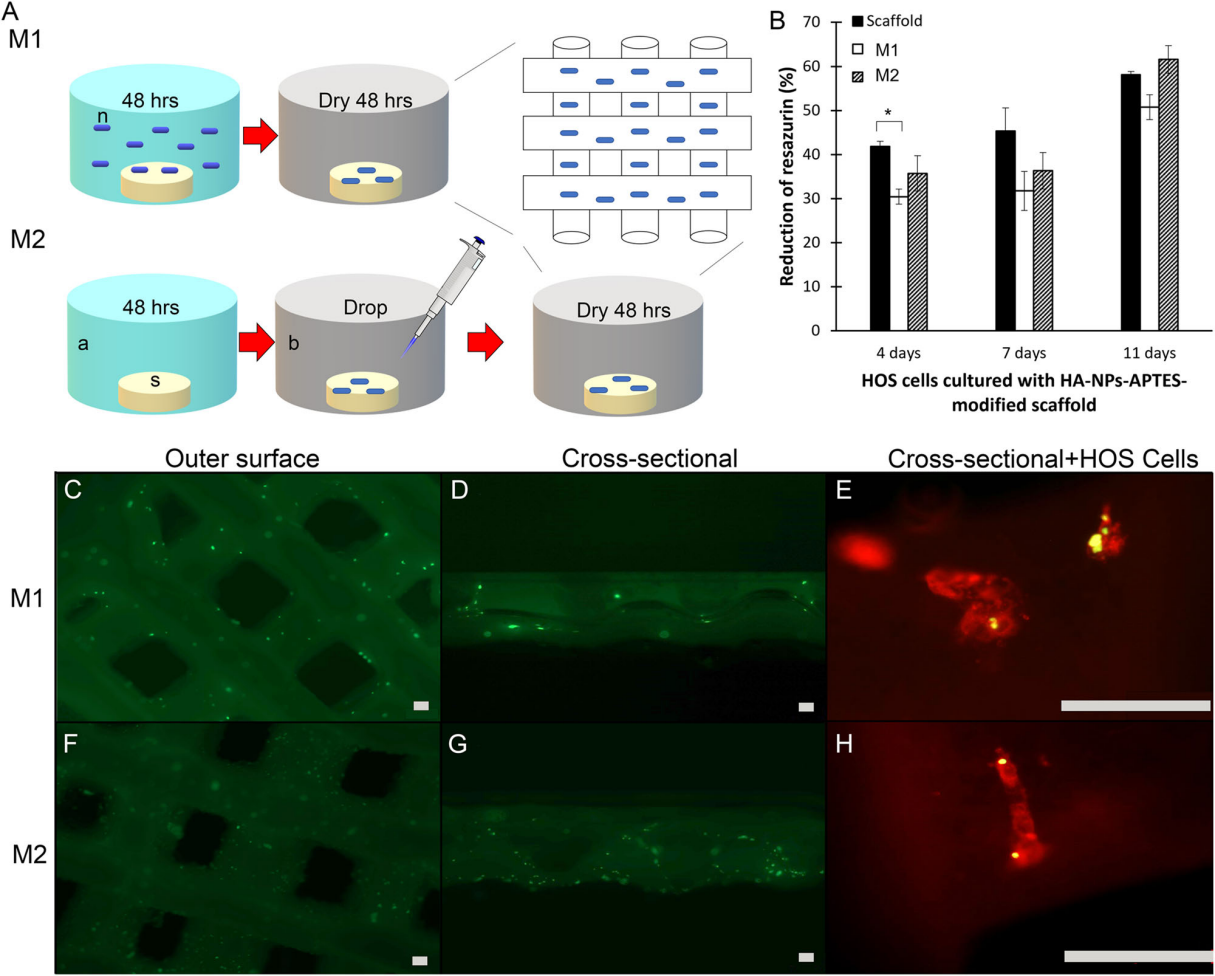
Modification of TCP/HA scaffold with HA-NPs-APTES. TCP/HA 3D printed scaffolds surface was modified by HA-NPs-APTES addition using two different methods. **A** Method1 (M1): TCP/HA 3D printed scaffolds were cured in sterile distillated water with HA-NPs-APTES in suspension. Method2 (M2), TCP/HA 3D printed scaffold were cured in sterile distillated water and dried before dropping HA-NPs-APTES suspension on their surface. (n = HA-NPs-APTES, s = Scaffold, a = distillated sterile water, b = dry culture plate). **B** HOS proliferation on bare TCP/HA scaffolds (Scaffold), scaffolds modified with HA-NPs-APTES using Method1 and 2 (M1 and M2) at day 4, 7, and 11 as measured by resazurin reduction. Means of 3 separate experiments in triplicate ± SD. Representative images of FITC labelled HA-NP-APTES repartition on scaffolds surface by using fluorescent microscopy (**C**, **D**) top view and cross-sectional view, method 1, (**F**, **G**) top view and cross-sectional view, method 2. using TCP/HA cement hardened by HA-NPs-APTES-suspended water. HOS cells were cultured on scaffolds prepared by method 1 (**E**) and Method 2 (**H**) and internalized HA-NPs-APTES-FitC. Scale bar = 100 µm. One-way ANOVA demonstrated significant differences of resazurin reduction in treated cells when compared to the control. *p < 0.05, **p < 0.01, ***p < 0.001.

The schematic figure of scaffold modification is shown in Fig. 1A.

#### Cell culture with TCP/HA scaffolds

With ethical approval (HREC-DCU 2022-038), human mandibular-derived osteoblasts (HmOBs) were cultured as described previously [25] using bone chips from third molars routine extractions. This study was performed in accordance with the Declaration of Helsinki. All participants and/or their legal guardians gave written informed consent. HmOBs from passage 5–8 were used in the following experiments. Human osteosarcoma cell lines HOS (CRL-1543, ATCC) and MG-63 (CRL-1427, ATCC) were also used. Briefly, all cells were cultured at 37 °C in a humidified atmosphere of 5% CO_2_ with Dulbecco’s Modified Eagle’s Medium with Glutamax (DMEM; Gibco® by Life Technologies, NY, USA) supplemented with 10% fetal bovine serum (FBS; Hyclone® Thermo scientific, Northumberland, UK) and1% penicillin-streptomycin-fungizone. The medium was replaced with fresh medium every 2 days. For biocompatibility assays, scaffolds were placed in 24-well plate before 3 × 10^4^ cells were seeded (day 0). Cells were allowed to adhere on the scaffolds for 24 h. On day 1, the scaffolds were transferred into a new culture well with fresh medium and further cultured for up to 21 days.

#### Resazurin assay

Resazurin assay was performed to examine cellular metabolic activity at day 4, 7 and 11 as described previously [31]. Briefly, resazurin dye solution (Sigma-Aldrich, MO, USA) in DMEM culture medium was prepared (0.1 μg/ml). Culture medium was removed, cells were washed once with PBS and 2 ml of resazurin medium were added and incubated for 4 h. Then, 200 µl of supernatants were collected, and optical density was measured at 570 and 630 nm to calculate the percentage of Resazurin reduction.

#### Fluorescent microscopy

HOS cells were plated on scaffold modified with FITC tagged HA-NPs-APTES in an 8-well culture slide (ibidi, Germany, Cat.No:80826), and cultured overnight. After 24 h incubation, cells were stained by 5 µg/ml FM® 4–64 lipophilic styryl dye (ThermoFicher scientific, MA, USA) on ice for 5 min then fixed with ice cold formalin 4% for 10 min. After 3 washes with Hanks’ balanced salt solution (HBSS; Invitrogen, CA, USA, Cat.No:14175079), slices were mounted with an aqueous mounting medium and observed on a Zeiss Axio Observer Z1 (Zeiss, Oberkochen, Germany) wide-field microscope.

#### Reverse transcription and quantitative polymerase chain reaction

On day 1 and day 6, total RNA was extracted by Trizol lysis reagent (Invitrogen, CA, USA,) according to manufacturer’s instructions. One microgram of total RNA was converted to cDNA by miScript II RT Kit (Qiagen, Hilden, Germany) on a thermal cycler (LifePro, Bioer, Hangzhou, China). For detection of miRNA, quantitative PCR was performed using Quantitect SYBR Green PCR Master mix (Qiagen, Hilden, Germany) on a PCR detection system (StepOnePlus, Applied Biosystem, CA, USA). The sequences of miRNA-302a-3p and RNU6-2 primers are shown in Table 1. The PCR settings were 95°C for 15 min followed by 40 cycles of amplification consisting of 94 °C for 15 s, 55 °C for 30 s, and 70 °C for 30 s. Relative quantification was assessed by the ΔΔCt method using miScript PCR controls RNU6-2 to normalize. Results were expressed as fold increase with respect to cells without treatment.

**Table 1:** Primer sequence.

Primer	Sequence	
	Forward	Reverse
miRNA-302a-3p	UAAGUGCUUCCAUGUUUUGGUGA	N/A
RNU6-2	GTGCTCGCTTCGGCAGCACA	N/A
COUP-TFII	CAAGGCCATAGTCCTGTTCACC	CGTACTCTTCCAAAGCACACTGG
RUNX2	CCGGAATGCCTCTGCTGTTATGA	ACTGAGGCGGTCAGAGAACAAACT
ALP	CGAGATACAAGCACTCCCACTTC	CTGTTCAGCTCGTACTGCATGTC
OCN	CTAGAGCGGGCCGTAGAAGCG	ATGAGAGCCCTCACACTCCTC
OSX	CGGGACTCAACAACTCT	CCATAGGGGTGTGTCAT
GAPDH	TCATGGGTGTGAACCATGAGA	GCTAAGCAGTTGGTGGTGCA

For detection of other genes, quantitative PCR was performed using a SensiFAST™ Kit (Meridian bioscience, MI, Italy, Cat.no BIO-82005) on PCR detection system (StepOnePlus, Applied Biosystem, CA, USA). Primer sequences for GAPDH, COUP-TFII, RUNX2, ALP, OCN, and OSX are shown in Table 1. The PCR settings were 95°C for 2 min, followed by 40 amplification cycles consisting of 95°C for 15 s, 60°C for 30 s. Reactions were performed in duplicate, and averages were used for analysis. Relative quantification was assessed by the ΔΔCt method using GAPDH to normalize. Results were expressed as fold increase with respect to cells without treatment.

### In vivo experiments

#### Animal procedures

All the animal experiment complied with Animal Research: Reporting In Vivo Experiments (ARRIVE) 2.0 checklist. The animal protocol was approved by the Chulalongkorn University laboratory animal center (Protocol no. 2173015). The required animal number is calculated using a power analysis to provide statistic power of 0.8 and type I error of 0.05. A total of 48 eight-week-old, male, C57/BL6 mice were acclimated for 2 weeks before surgeries. Mice were randomly assigned into 4 groups (*n* = 4 each). First, no scaffold (Control). Second, mice received bare scaffold (Scaffold). Third, mice received TCP/HA scaffold with HA-NPs-APTES without miRNA (Scaffold+HA-NPs-APTES). Fourth, mice received TCP/HA scaffold with HA-NPs-APTES-miRNA-302a-3p (Scaffold+HA-NPs-APTES-miR). To create the critical size defects, mice were intraperitoneal injected with Tiletamine-zolazepam (Zoletil®; 20 mg/kg) and Xylazine (2 mg/kg). Ocular lubrication (Vidisic® gel) was applied to prevent corneal drying during anesthesia. Enrofloxacin (5 mg/kg) and Carprofen (5 mg/kg) were given subcutaneously for antibiotic prophylaxis and analgesic respectively. Before any intervention, anesthetic depth was assessed by tail pinching with firm pressure, the animal also had to show loss of responses to reflex stimulation. After that, local anesthesia was given at scalp by subcutaneous injection with 0.1 ml of 2% mepivacaine with 1:100,000 epinephrine (Scandonest®, Septodont USA). After that, the scalp was wiped with 70% ethanal and then iodine to sterile to operation field. One cm incision line was created in the middle of the scalp with surgical blade no. 15 then a 4 mm diameter round defect was created using trephine bur on the left side of the calvarium. Scaffold were placed in the defect according to the allocation strategy described above without fixation then the site was sutured so that the skin retention ensured scaffolds primary stability. All mice were monitored and weighed daily. The mouse was excluded and euthanized if the weight reduced by more than 15% in 7 days. At 2-, 4- and 6-weeks post-surgery, mice were sacrificed by exposure to carbon dioxide and the samples were collected for micro-computed tomography and histological analysis.

#### Micro-computed tomography (micro-CT)

After sacrifice, skulls were collected and fixed in paraformaldehyde 4%. Bone growth was evaluated using micro-CT35 from Scanco Medical, Switzerland. Scans were performed for 2375 mSec at 70 kV, with an image pixel size of 16.259 µm. Three-dimensional reconstructions and analysis were performed using the analysis software from Scanco Medical (SCANCO Medical AG, Switzerland). The analyzer was not aware of the group of the sample while analyzing. The bone volumes relative to tissue volumes (BV/TV) were determined within the scaffold area. Whole pores were enumerated (top view) and a ratio unfilled by new bone vs total was calculated.

#### Histological analysis

After micro-CT analysis, samples were decalcified and embedded in paraffin. Blocks were cut in sagittal plane through the center. Six continuous sections (5 μm each) from the middle of the defect toward defect border were then prepared and mounted on glass slides before staining with hematoxylin and eosin (H&E) or Masson’s Trichrome to assess for bone formation. For Histomorphometry, histological sections were scanned on a KEYENCE VHX-6000 digital microscope (KEYENCE International, Belgium). The scaffolds were divided into 4 parts (1 mm wide) from left to right margin (Fig. 5B). Three zones were then defined: 1- center zone including 1 mm to the left and 1 mm to the right from the scaffold central point and 2–3 the border zones including left and right first mm from the scaffold. The analyzer was not aware of the group of the sample while analyzing. The percentage of new bone formation to total area in each part was quantified by using the KEYENCE software.

#### Statistical analysis

The data were shown as the mean values ± standard deviation or standard error of mean. Data normal distribution was verified by using a Shapiro-Wilk test. Homogeneity of variances was analyzed before a one-way ANOVA followed by a Tukey post hoc test was performed by the SPSS v 21.0 statistical software package. The difference was considered statistically significant when the *p* value was inferior to 0.05.

## Results

### TCP/HA scaffold-HA-NPs-APTES effect on bone cells in vitro

Before cell culture, we first verified that HA-NPs-APTES were adsorbed on TCP/HA scaffolds surface either modified by using Method 1 or 2 (M1-2) (Fig. 1A) with fluorescent HA-NPs-APTES-FITC. By using confocal fluorescent microscopy, we observed HA-NPs-APTES equally dispersed on superficial (Fig. 1C, F) and deep layers (Fig. 1D, G) of both M1 and M2 scaffolds.

TCP/HA scaffolds were then assessed for biocompatibility. HOS cells were cultured on bare scaffolds, M1, and M2 scaffolds for up to 21 days. Cellular uptake of HA-NPs-APTES was verified after 24 h (Fig. 1E, H). Initially, cell proliferation on M1 scaffold was significantly lower than controls by approximately 25% as measured by resazurin assay (Fig. 1B) [31]. However, a gradual increase of cell proliferation was observed after day 7, and that was comparable to control values at 21 days (Supplementary Fig. 1).

### TCP/HA scaffold-HA-NPs-APTES delivers miRNA-302a-3p efficiently in vitro

**Fig. 2:**
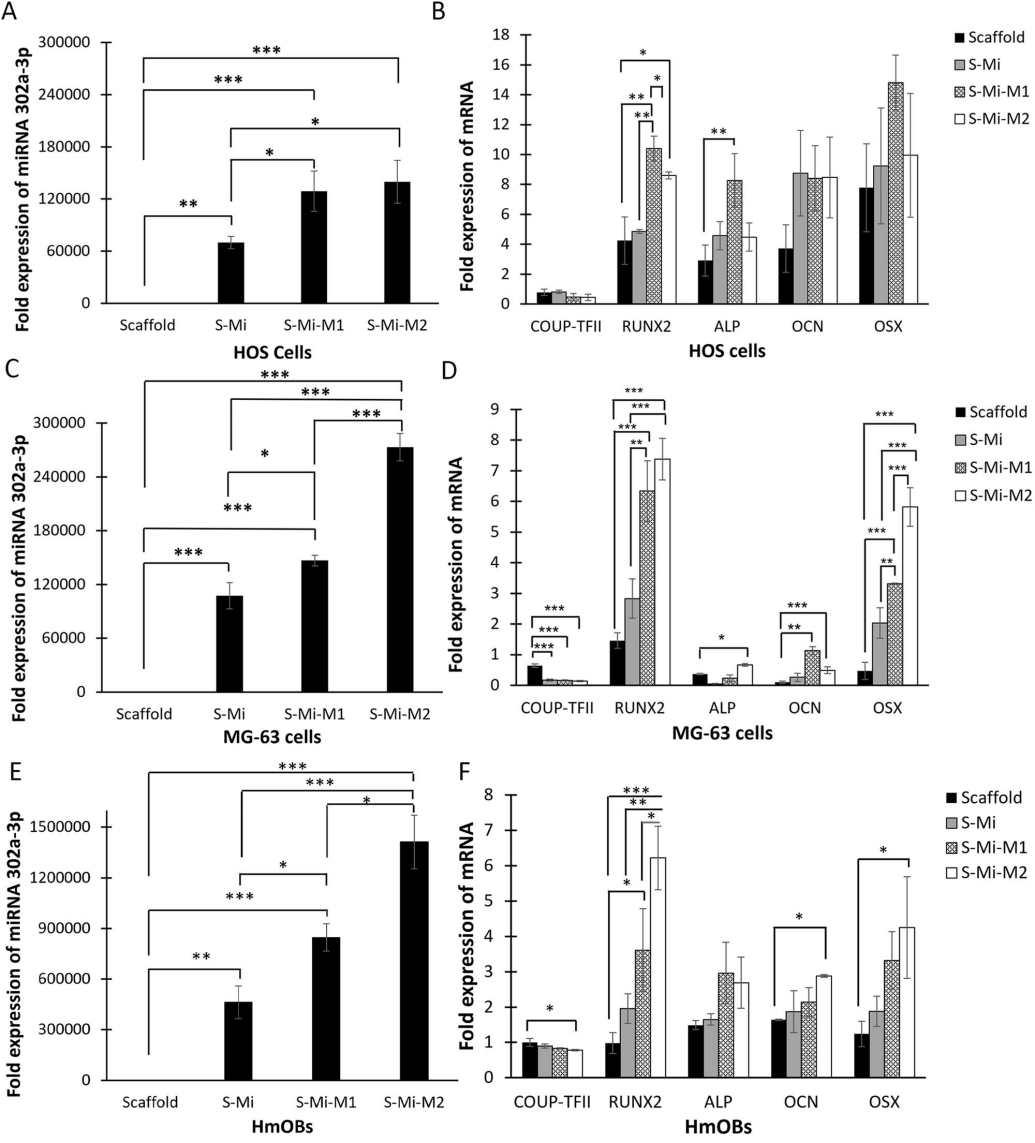
Delivery of miRNA-302a-3p by HA-NPs-APTES-modified 3D-printed-TCP/HA scaffolds and subsequent effect on osteogenic genes expression. HOS, MG63 and HmOBs were cultured for 6 days on bare scaffolds (Scaffold), scaffolds directly conjugated with miRNA (S-Mi) or modified with HA-NPs-APTES-miR-302a-3p according to Method 1(S-Mi-M1) or Method 2 (S-Mi-M2), before RNA extraction and qRT-PCR for MiRNA-302a-3p, COUP-TFII, RUNX2, ALP, OCN and OSX. MiRNA-302a-3p expression in HOS (**A**), MG-63 (**C**), and primary human osteoblast (HmOBs) cells (**E**). Results expressed as fold increases vs miRNA-302a-3p expression in cells grown on culture plate and normalized to RNU6-2. COUP-TFII, RUNX2, ALP, OCN and OSX gene expression in HOS (**B**), MG-63 (**D**) and HmOBs (**F**). Results expressed as Fold increases vs target gene expression in cells grown on culture plate and normalized to GAPDH. Mean ± SD, *p < 0.05, **p < 0.01, ***p < 0.001.

MG63, HOS and HmOBs were cultured on bare scaffolds, scaffolds directly conjugated to miRNA-302a-3p (S-Mi), and scaffolds modified by HA-NPs-APTES and miRNA-302a-3p according to M1 and M2 (S-Mi-M1 or M2) to assess for the highest and most effective miRNA-302a-3p delivery affecting target genes. A significant increase of miRNA expression was observed in all cell types after 6 days of culture with all scaffolds with any modification (Fig. 2A, C, E), demonstrating the success of miRNA delivery.

Within the same cell type, miRNA expression by M1 (*p* < 0.05) and M2 (*p* < 0.001) scaffolds was higher than scaffolds directly conjugated with miRNA (S-Mi). Except for HOS, M2 proved higher deliveries than M1 that were translated by a doubling in miRNA-302a-3p expression.

Next, the downstream effect of miRNA-302a-3p upregulation was investigated. The expressions of target genes, including COUP-TFII, RUNX2, and other osteogenic genes (ALP, OCN and OSX), were analyzed on day 6 after miRNA delivery (Fig. 2B, D, F). miR-302a-3p upregulation caused a significant COUP-TFII gene downregulation in MG63 (Fig. 2D) and only with the M2 scaffolds in HmOBs. (Fig. 2F), but not in HOS cells (Fig. 2B). Simultaneously, RUNX2 expression was significantly increased in all cell types after miR-302a-3p upregulation, especially with the M2 scaffolds in MG63 cells (Fig. 2D) and HmOBs (Fig. 2F).

In MG63 cells (Fig. 2D), the increase of miR-302a-3p expression led to approximately 5-fold downregulation of COUP-TFII after miRNA delivery by S-Mi, S-Mi-M1 and S-Mi-M2 scaffolds. Consistently, RUNX2 expression was increased by 6.34 ± 0.99 folds (*p* < 0.001), and 7.38 ± 0.68 folds (*p* < 0.001) in cells cultured on S-Mi-M1 and S-Mi-M2, respectively, but not in cells with S-Mi. ALP expression was significantly upregulated only in cells with S-Mi-M2. Agreeing, OCN and OSX were upregulated in cells with S-Mi-M1 and M2, but not S-Mi.

In general, a similar osteogenic gene profile was observed in HOS and HmOBs after miRNA delivery, despite a lack of significance in most of the conditions observed, except for RUNX2. M2 consistently demonstrated upregulation of osteogenic genes higher than other modification methods. However, scaffold with miRNA without nanoparticle (S-Mi) showed no significant gene expression than scaffold alone in most of the conditions therefore, we didn’t include it as a control in the in vivo model.

### TCP/HA scaffold modified with HA-NPs-APTES-miRNA promote bone regeneration in critical-sized calvarial defects in mice

Based on the in vitro data, the scaffolds modified with HA-NPs-APTES-miRNA-302a-3p using M2 were used for the in vivo experiments. Mice were assigned into 4 groups (*n* = 4 in each group): 1- no scaffold (Control), 2- bare scaffold (Scaffold), 3- scaffold+HA-NPs-APTES without miRNA (Scaffold+HA-NPs-APTES), and 4- scaffold +miRNA-302a-3p-conjugated with HA-NPs-APTES and miRNA (Scaffold+HA-NPs-APTES-miR). Calvarial defects were filled according to these groups and defects healing was analyzed at week 2, 4, and 6 after placement by micro-CT and histological analysis. All mice survived and met inclusion criteria.

**Fig. 3:**
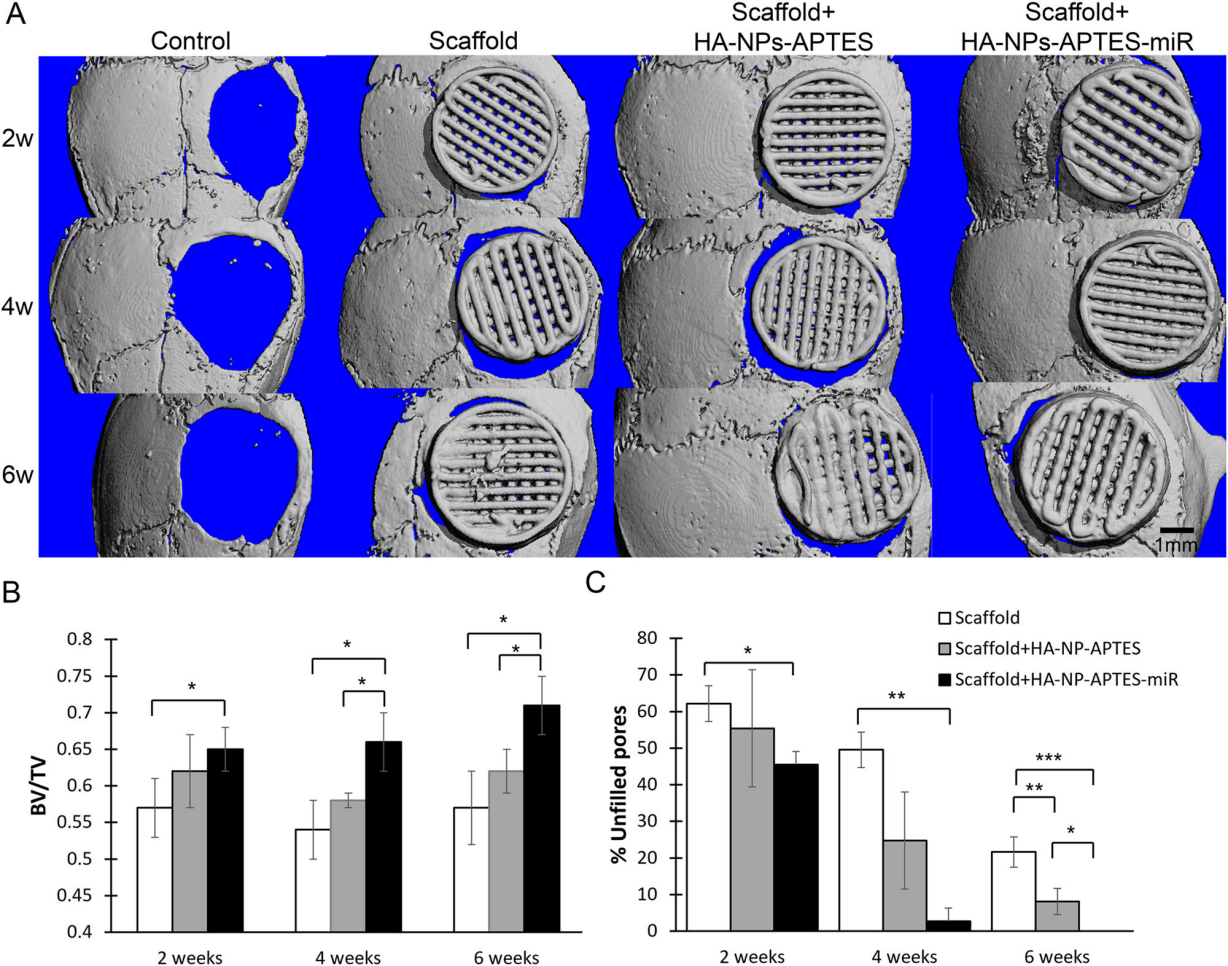
Scaffolds engraftment in a critical-sized bone defect on mouse calvarium as assessed by microCT analysis. Bare scaffolds (Scaffold), Scaffold-associated HA-NPs-APTES according to Method 2 (Scaffold+HA-NPs-APTES) and Scaffold-associated HA-NPs-APTES according to Method 2 + miRNA-302a-3p (Scaffold+HA-NPs-APTES-miR) were placed in critical-sized bone defects on mouse calvarium for 2, 4 and 6 weeks (controls: defects left empty), *n* = 4 in each group. **A** Representative micro-CT images. Scale bar = 1 mm. **B** Bone volume per tissue volume within the entire scaffold area. **C** Percentage of unfilled scaffolds pores to total pore number. *p < 0.05, **p < 0.01, ***p < 0.001.

As shown by micro-CT analysis, the defects in the control group failed to heal spontaneously as shown at 2–4 and 6 weeks (Fig. 3A) and was therefore considered as a critical-sized defect.

At 2 weeks post placement, we observed a trabecular bone volume to total volume fraction (BV/TV) of 0.566 ± 0.042 in defects filled with scaffolds alone (Fig. 3B), 0.062 ± 0.049 with Scaffold+HA-NPs-APTES and 0.653 ± 0.034 with scaffold + HA-NPs-APTES-miRNA (p < 0.05 when compared to scaffold alone). These ratios were not modified in control conditions (Scaffold alone and Scaffold+HA-NPs-APTES) after 4 and 6 weeks (Fig. 3A) when it gradually and significantly increased with HA-NPs-APTES-miR to 0.656 ± 0.042 and 0.714 ± 0.037, respectively.

We also enumerated the number of unfilled pores on the scaffolds on micro-CT scans and calculated the percentage of unfilled pores to total pore number (74 pores initially) (Fig. 3C). At 2 weeks, the % unfilled pores on Scaffold+HA-NPs-APTES-miRNA (45.5 ± 4.1) was significantly lower than scaffold alone and Scaffold+HA-NPs-APTES (62.16 ± 4.9 and, 55.41 ± 4.9). At 4 weeks, unfilled pores on Scaffold+HA-NPs-APTES-miRNA were rarely seen (2.7 ± 2.58), while 49.55 ± 16.01 unfilled pores in the scaffolds alone, 24.77 ± 13.26 in Scaffold+HA-NPs-APTES could be observed. At 6 weeks, there were no unfilled pores left in scaffold modified with HA-NPs-APTES-miR, 8.11 ± 3.58 in scaffolds + HA-NPs-APTES, and 21.62 ± 3.6 in scaffold alone.

**Fig. 4:**
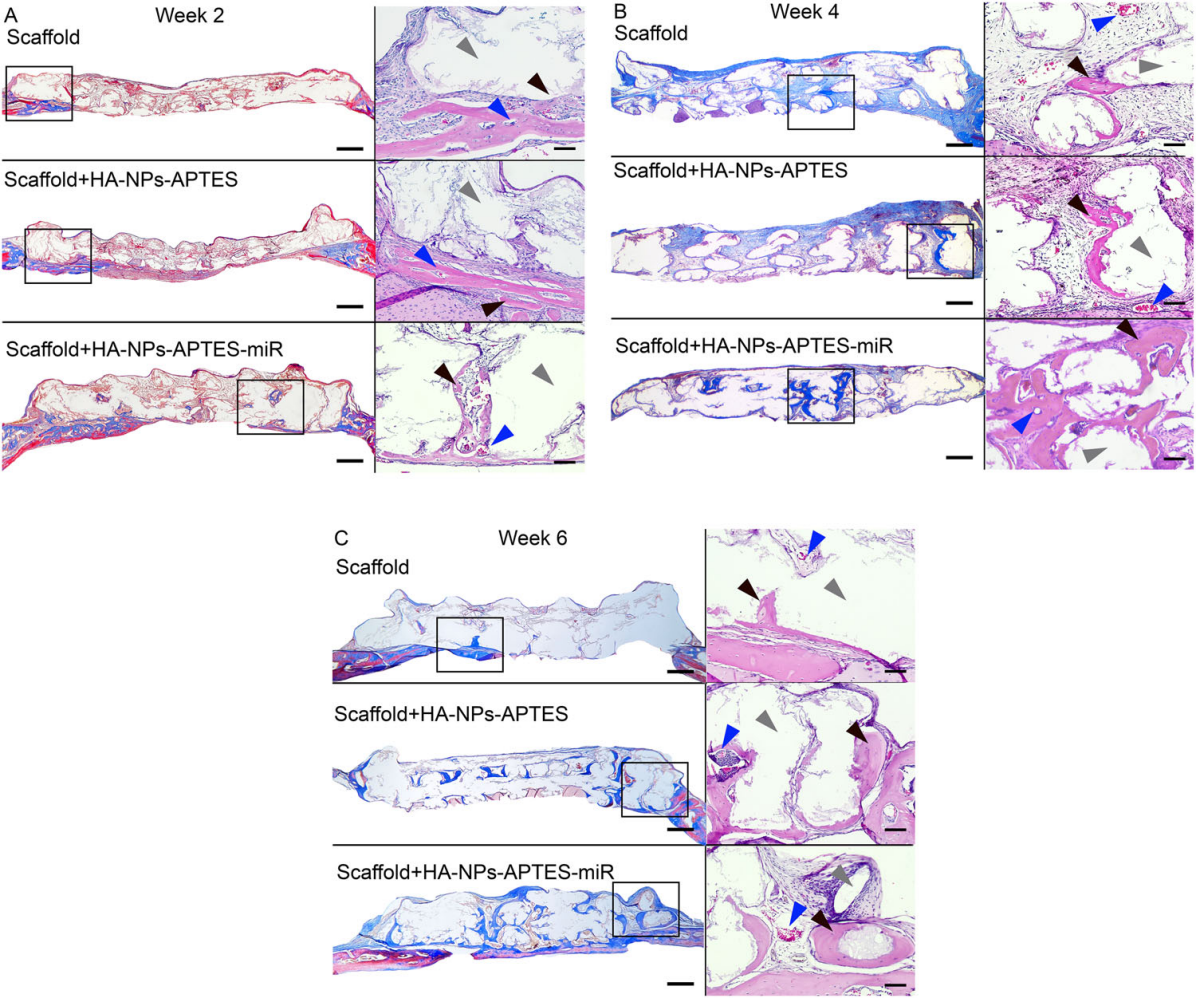
HA-NPs-APTES-miRNA-302a-3p enhances new bone and blood vessel growth. Bare scaffolds, Scaffold-associated HA-NPs-APTES according to Method 2 (Scaffold+HA-NPs-APTES) and Scaffold-associated HA-NPs-APTES according to method 2 + miRNA-302a-3p (Scaffold+HA-NPs-APTES-miR) were placed in critical-sized bone defects on mouse calvarium for 2, 4- and 6-weeks, *n* = 4 in each group. Slides shown are representative histological sections at (**A**) 2 weeks, (**B**) 4 weeks, and (**C**) 6 weeks. Slides were cut in sagittal plane in the middle of scaffolds. Masson’s trichrome staining showed new bone formation in dark blue. Scale bar = 250 µm. Some areas were magnified (within the rectangle window) and observed with a H&E staining. Black arrow = new bone; Blue arrow = blood vessel; Gray arrow = scaffold; Scale bar = 50 µm.

**Fig. 5:**
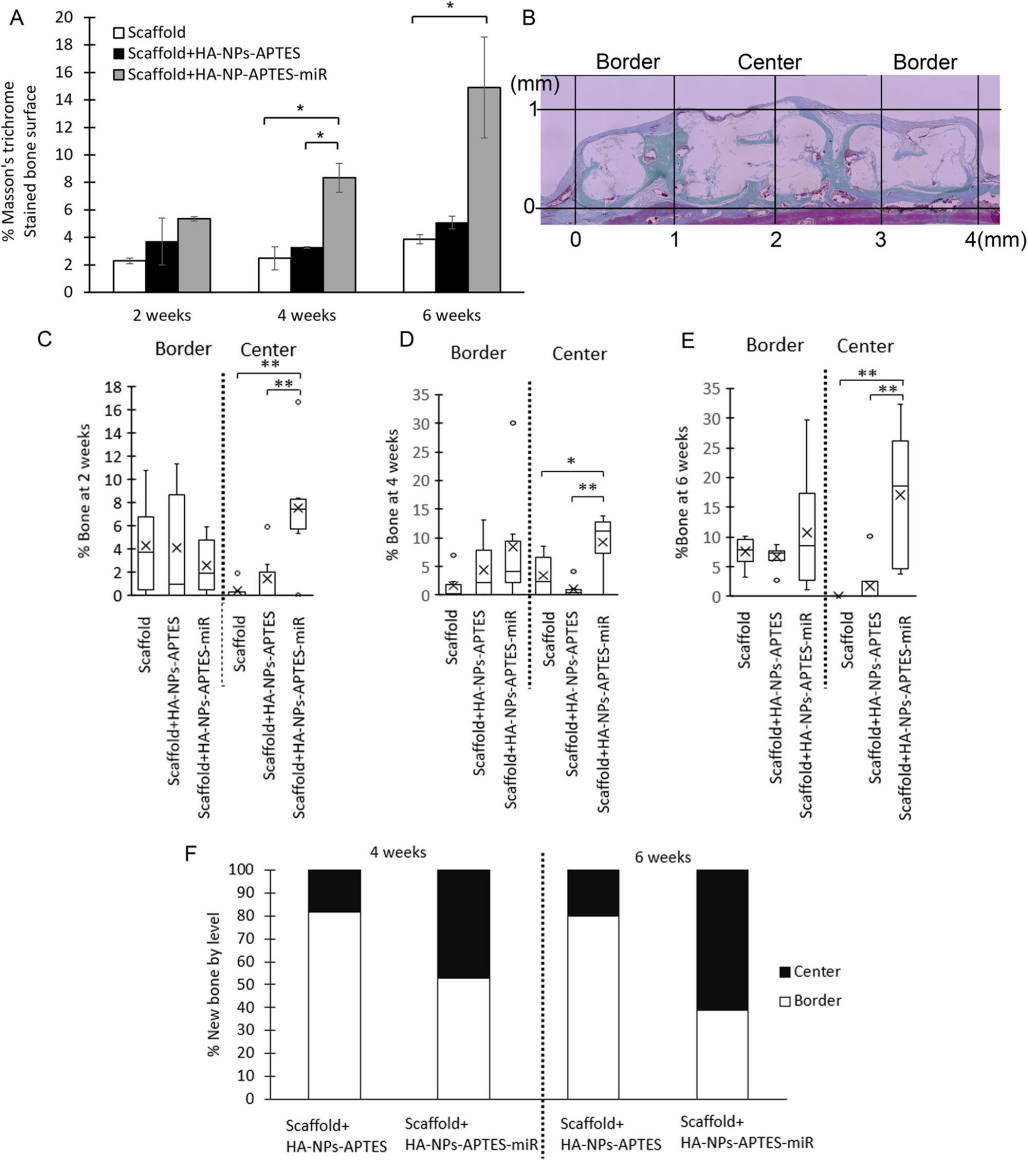
Histomorphometrical evaluation of new bone surfaces. Histological sections were stained with Masson’s trichrome, digitalized and the new bone tissue was quantified within the entire scaffold surface (**A**). The scaffold (diameter = 4 mm) was divided into a border region (0–1 mm and 3–4 mm) and a center region (1–3 mm) for each sample (**B**). New bone quantification in each region at (**C**) = 2 weeks, (**D**) = 4 weeks, (**E**) = 6 weeks. Boxes: 25-75%, x: mean, Horizontal line: median, white dot: outlier. The relative proportion of new bone within center or border regions was summarized in (**F**), *n* = 4 each group.

Scaffolds engraftment was visible in all conditions at 2 weeks (Fig. 4A), essentially in contact with peripheral calvarial bone margins from where few new bone sprouts were observed and started the osseointegration process. These areas were also filled with fibro-conjunctive tissue rich in capillaries. In scaffolds modified with HA-NPs-APTES-miRNA, new bone growth was more advanced and started migrating towards the construct center. Histomorphometrically, we could not detect difference in terms of total new bone within the entire scaffold surface, which represent 2.29 ± 0.21, 3.7 ± 1.7 and 5.35 ± 0.15 for bare Scaffold, Scaffold+HA-NPs-APTES and Scaffold+HA-NPs-APTES-miRNA, respectively (Fig. 5A). After segmenting the histological sections into 2 zones (Fig. 5B), border (0–1 mm from the periphery i.e. 0–1 and 3–4 mm on a plane projection) and center (central zone from 1 mm from the periphery or between 1–3 mm on a a plane projection), a significant difference was observed in the center region were new bone reached a mean of 7.5 ± 2.7% in Scaffold+HA-NPs-APTES-miR vs. 1.41 ± 1.2% and 0.38 ± 0.3% in Scaffold+HA-NPs-APTES and bare scaffold, respectively (Fig. 5C).

At 4 weeks after implantation, the osteogenic process was initiated in the entire scaffolds surface in all conditions, i.e. an osteoid tissue rich in capillaries and foci of mineralization were visible from border to center (Fig. 4B). In some areas, the scaffold material was perfectly osseointegrated. We observed largely more mineralized tissue in Scaffold+HA-NPs-APTES-miRNA, from border to center, with respect to controls (bare Scaffold and Scaffold+HA-NPs-APTES). Total new bone represented 8.3 ± 1.05% of the surface in Scaffold+HA-NPs-APTES-miR which was significantly more than 3.26 ± 0.7% in Scaffold+HA-NPs-APTES and 2.48 ± 0.85% in bare scaffolds (Fig. 5A). The new bone was equally distributed in border regions in all conditions (1.62 ± 1.36, 4.43 ± 2.79 and 8.46 ± 5.59). The difference recorded with total new bone was observed only in center regions where 9.25 ± 2.67% of the surface was filled with new bone in Scaffold+HA-NPs-APTES-miR vs. 1 ± 0.79% and 3.4 ± 1.97% in Scaffold+HA-NPs-APTES and bare scaffolds, respectively (Fig. 5D).

At 6 weeks, the process of bone growth and scaffold integration continued according to same kinetic and ratios leading to large filling of scaffolds pores on the entire surface in Scaffold+HA-NPs-APTES-miR. Bone tissue had matured and turned to lamellar bone with numerous osteocyte niches. The difference with the surrounding calvarial bone tissue was not visible anymore and continuity was evident. Same observations were done in control conditions (Scaffold+HA-NPs-APTES and bare scaffolds). Despite large portions of scaffolds osseointegrated and strong vascularization, we could not observe any sign of material resorption (Fig. 4C). In Scaffold+HA-NPs-APTES-miR, the total new bone now filled 14.9 ± 3.65% of the surface when the growth was largely slower in Scaffold+HA-NPs-APTES and bare scaffolds with 5.08 ± 0.46 % and 3.86 ± 0.33 filling. Border regions were filled in the same proportions (ca. 10%) in all conditions. New bone occupied 17 ± 5.77% of the center region in Scaffold+HA-NPs-APTES-miR, a proportion significantly higher than the ca. 2% observed in Scaffold+HA-NPs-APTES and bare scaffolds (Fig. 5E). The relative proportion of new bone within the center region of Scaffold+HA-NPs-APTES-miR evolved from 50 to 60% between 4 and 6 weeks, reflecting large osseoconduction and osseoinduction of this modified construct when compared to the non-modified Scaffold+HA-NPs-APTES where the central region represented only 20% of the total filling both at 4 and 6 weeks (Fig. 5F).

## Discussion

Osteoinductive materials that stimulate stem cell differentiation and enhance bone regeneration are important for treating critical-sized bone defects. This study demonstrated an efficient delivery of miRNA-302a-3a by HA-NPs-APTES-adsorbed on 3D-printed TCP/HA scaffold in both in vitro and in vivo model. The miRNA-302a-3a was conjugated with HA-NPs-APTES before being adsorbed onto the surface of TCP/HA scaffolds for delivery. The 3D-printed TCP/HA scaffolds that we used as a basis for modifications was shown to be highly osteoconductive due to its regular architecture. By adding the bioactive miRNA-302a-3a, we successfully extended the TCP/HA regenerative system toward essential osteoinductive properties.

HA-NPs were used to deliver miRNA intracellularly. The NP size and morphology affects the cellular uptake [32]. Here we used rod-shaped NP with the length of 100-150 nm and width of 10 nm whose efficacy was already demonstrated, especially APTES treatment that improve the amount and stability of attached miRNA [20, 25]. APTES is an amino silane used for silane functionalization of HA-NPs surfaces [33]. APTES-modified particles were described as non-cytotoxic [34]. We confirmed this status in vitro in a series of experiment by using osteoblastic cell lines [20, 25]. Current data on TCP/HA scaffolds modified with HA-NPs-APTES also confirmed biocompatibility. Efficient delivery of miRNA-302a-3a by HA-NPs-APTES adsorbed on TCP/HA scaffolds, its innocuity and action on target genes was also demonstrated, as shown by cell proliferation assays, internalization assays by using FITC tagged nanoparticles and relative osteogenic gene expression assays.

Regarding the method for modifying TCP/HA scaffold surface by HA-NPs-APTES adsorption, we used two different approaches: M1 that incorporated HA-NPs-APTES-miRNA-302a-3a into the cement during the hardening process and M2 which absorbed HA-NPs-APTES-miRNA-302a-3a after the cement setting. The latter was more efficient, probably due to lower nanoparticles adsorption and further facilitated release when compared to M1 where nanoparticles were probably deeply incorporated in the surface cement and thus less accessible to cells. This is the reason why we used M2 for the rest of the study. How these different approaches influence the efficiency of miRNA delivery to target cells is not within the scope of this study but remains to be further investigated.

For in vivo studies, we used a mouse calvarial model. The defects could not be regenerated without bone graft, as shown by the controls without scaffold, and were therefore considered critical in size.

The 3D printed TCP/HA scaffold that we used as a basis for modifications was thoroughly characterized by our group and was shown to be highly osteoconductive due to its regular architecture [7, 28–30]. Scaffold modified by M2 significantly facilitated bone healing in the critical-sized defects in vivo. With miRNA-302a-3a, new bone formation reached the central area of the defect at 2 weeks when it remained in defect margins without miRNA-302a-3a. Bone quantity was also largely improved by miRNA-302a-3p addition. The same profile was observed after 6 weeks of healing. These results therefore demonstrated miRNA-302a-3p as a bioactive molecule. Moreover, it can be easily combined to HA-NPs-APTES and delivered in vivo once absorbed on scaffold surface. Finally, miRNA-302a-3p can implement osteoinductive properties to 3D-printed TCP/HA scaffolds.

The 3D scaffolds of TCP/HA with modification have been reported to promote bone repair in critical-sized defects. The positive results of the BMP2-coated TCP/HA scaffolds implanted into mouse calvarium defect were shown after 8 weeks [35, 36]. Another model used collagen-coated β-TCP scaffolds for a slow release of plasmid DNA encoding miR-200c and facilitate healing of a critical-sized defect in rat calvarial bone at 4 weeks after implantation [36]. In this study, we predicted that new bone formation could be saturated within the TCP/HA scaffold after 8 weeks, the differences between Scaffold+HA-NPs-APTES and Scaffold+HA-NPs-APTES-miR reflecting the benefits of adding miRNA as an active molecule was best shown at early time point. Therefore, an enhancement of bone regeneration by the miRNA-302a-3p on our bone regenerative system was shown as early as 2,4, and 6 weeks after implantation. Thus, the scaffold modified with HA-NPs-APTES-miR accelerates the bone healing process in a critical-sized defect of a mouse calvarial model.

The miRNA-302a-3p is not only involved in bone regeneration but may also inhibit cell migration and proliferation in some cancers [37–39], negatively regulate endothelial inflammatory responses via the NF-κB pathway [40]. In addition, miRNA-302a was detected in exosomes derived from stem cells suggesting its contributing role in systemic and comprehensive biological network [41]. As it may affect other cell or tissue function, it should be used in a very controlled way. Although, miRNA-302a-3p was known to repress angiogenesis in trophoblast [42] and oxidative induced endothelial cell line by targeting VEGFA [43]. However, a recent study showed that VEGFA from osteoblasts may not always be required for the bone formation in all scenario [44], this shows the complexity of the phenomenon. Therefore, it would be interesting to explore more on angiogenic gene expression in osteoblast received miRNA-302a-3p. However, it is beyond the scope of this research.

Previous studies have considered a variety of approaches associated with scaffolds, stem cells, gene therapy, and cellular signals to gain proper mechanical property and osteoinduction for bone tissue engineering [45]. Although the use of mesenchymal stem cells can successfully mediate osteoinductive healing, some microenvironments such as inflammation, aging, and estrogen-deficient conditions could impact on its inductive functions [46]. This study demonstrated the alternative cell-free approach of the HA/TCP scaffold with an advancement from miRNA function to draw benefits like mesenchymal cell-based approaches during bone regeneration. The effects described to date, in addition to bone regeneration, are quite beneficial, but possible side effects cannot be excluded. The presence of HA-NPs-APTES in adjacent and more distant tissues remains to be verified.

## Conclusions

This study demonstrated that the TCP/HA scaffold modified with HA-NPs-APTES facilitated delivery of miRNA and enhanced osteoinduction of the grafting scaffold. The 3D-printed scaffold allowed a customized shape designed from radiographic images, with osteoinductive property from the HA-NPs-APTES-miRNA-302a-3P, this could be promising for future clinical application.

### Supplementary information

Supplemental Material

## Data Availability

The datasets generated and/or analyzed during the current study are available from the corresponding author upon request.
