# Supplementary material for: 3D-printed TCP-HA scaffolds delivering MicroRNA-302a-3p improve bone regeneration in a mouse calvarial model
Source: BDJ Open. 2023 Nov 24;9:50. doi: 10.1038/s41405-023-00177-1 (PMC10673873; doi:10.1038/s41405-023-00177-1)
Supplement: Supplementary file 1 — Supplemental Material [file 41405_2023_177_MOESM1_ESM.pdf]

Supplementary figure 1

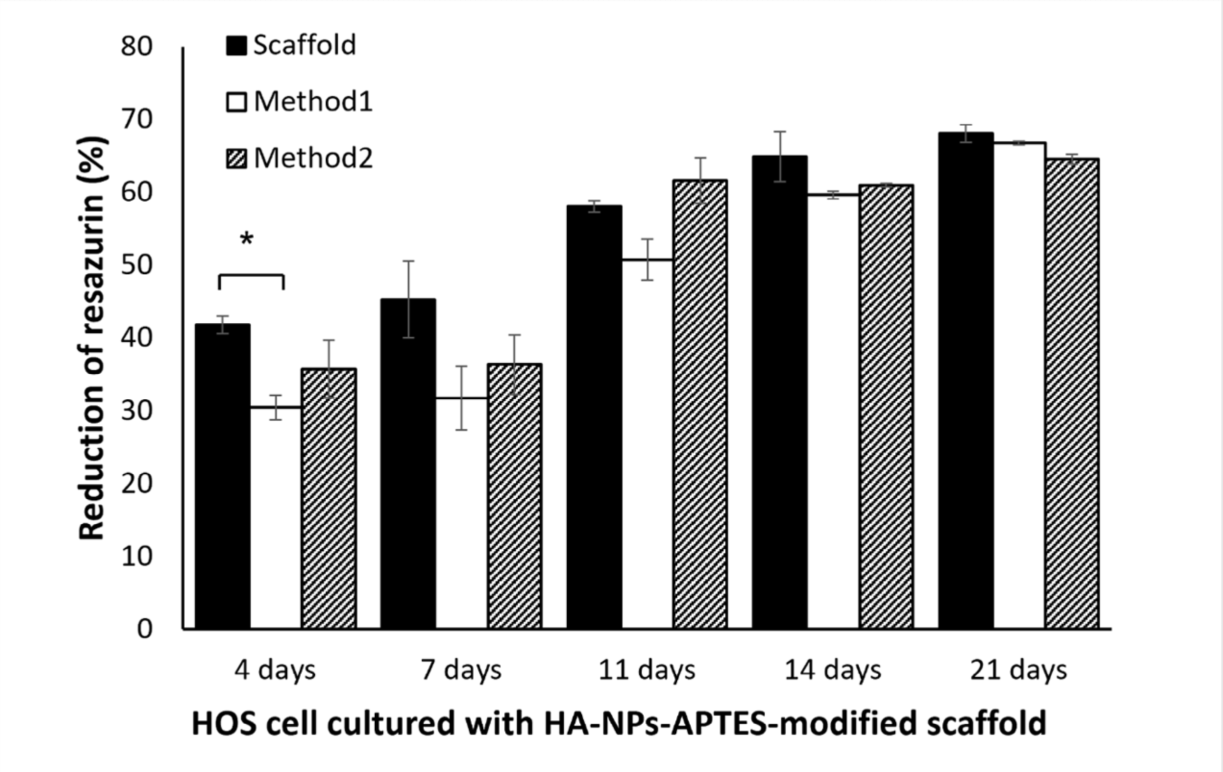

Resazurin reduction of HOS on TCP/HA scaffold modified with HA-NPs-APTES. Percent resazurin reduction in HOS cultured on TCP/HA scaffold modified with HA-NPs-APTES on day 4, 7, 11, 14 and 21 days. Bars represent means from 3 replicates with standard deviation. One-way Anova demonstrated significant differences of resazurin reduction in treated cells when compared to the control. *: $p<0.05$,

Supplementary figure 2

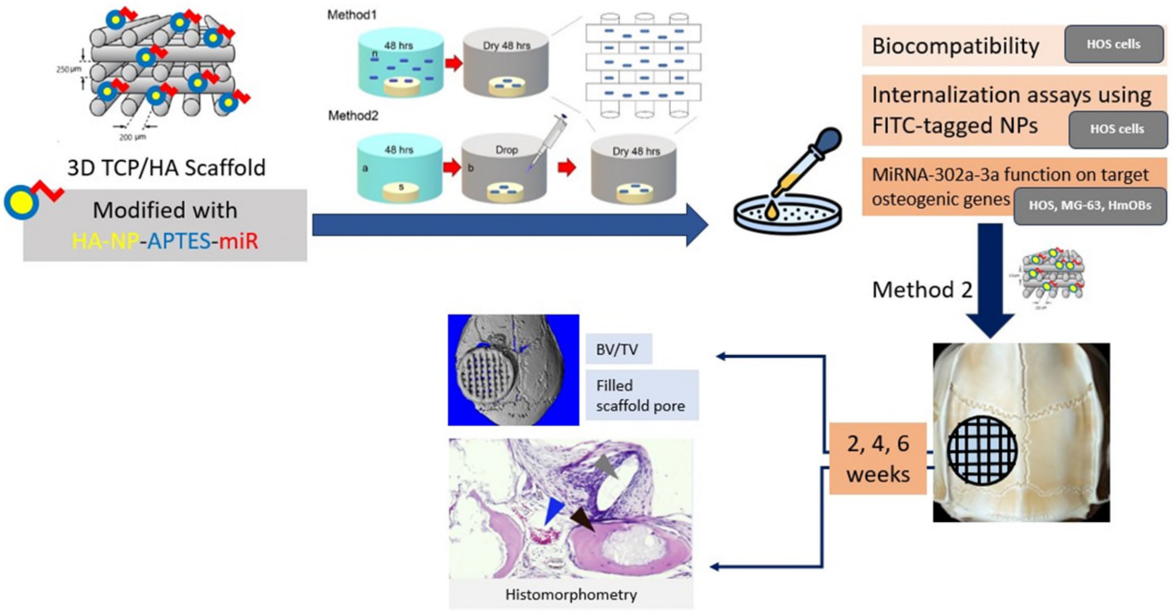

Experiment design illustration
